# Biodex© training post-stroke for postural stability in the upper trunk: A pilot study

**DOI:** 10.4102/sajp.v76i1.1416

**Published:** 2020-09-30

**Authors:** Helena W. Nel, Witness Mudzi, Elizabeth C. Janse van Vuuren, Eustasius Musenge

**Affiliations:** 1 Department of Physiotherapy, Faculty of Health Sciences, University of the Witwatersrand, Johannesburg, South Africa; 2 Department of Physiotherapy, School of Health and Rehabilitation Sciences, Faculty of Health Sciences, University of the Free State, Bloemfontein, South Africa; 3 Post-graduate School, University of the Free State, Bloemfontein, South Africa; 4 Department of Biostatistics, School of Public Health, University of the Witwatersrand, Johannesburg, South Africa

**Keywords:** Biodex©, postural stability, upper trunk postural stability, upper limb function, stroke survivors

## Abstract

**Background:**

Stroke affects upper trunk postural stability and upper limb function in approximately 85% of stroke survivors. Upper trunk postural stability is essential for functioning of the upper limb and is a prerequisite for hand function. The rehabilitation of the upper limb and upper trunk post-stroke remains a challenge because of poor recovery of motor and sensory function.

**Objectives:**

To determine the effect of Biodex© upper limb weight-bearing training on upper trunk postural stability in patients post-stroke.

**Method:**

A longitudinal randomised control pilot trial with single blinding was undertaken to assess postural stability on the Biodex© at baseline and 1-month post-baseline. In addition to standard rehabilitative care, upper limb weight-bearing training on the Biodex© was added for participants in the experimental group. Descriptive data analysis and the Mann–Whitney test for group comparisons were done using STATA (*p* < 0.05).

**Results:**

Fifteen participants took part, seven in the control and eight in the experimental group, with an overall median age of 55 years. At baseline there were statistically significant lower scores in the experimental group on overall (*p* = 0.02) and anterior/posterior (*p* = 0.009) stability level 6 (moderately unstable base of support) in the upper trunk postural stability scores. No statistically significant improvements were noted between groups on any of the Biodex© stability levels at 1-month post-baseline testing (*p* > 0.05).

**Conclusion:**

Upper limb weight-bearing training with the addition of Biodex© training did not result in improvements in upper trunk postural stability.

**Clinical implications:**

The findings suggest that exercising on a moderately unstable base of support may improve upper trunk postural stability in patients post-stroke. The addition of Biodex© training to standard rehabilitative care for retraining and exercising upper trunk postural control in a weight-bearing position does not lead to better outcomes than standard care.

## Introduction

After a stroke, approximately 85% of survivors present with an initial motor and/or sensory deficit of the upper limb and complications may arise from these deficits (Lang et al. 2012; Morris et al. 2013). Improvement in upper limb function is poor and 55% – 75% of cases still present with poor upper limb function 3 to 6 months after the initial incident (Harris et al. 2010). The upper limb plays an important role in activities of daily living (ADL), as the execution of normal ADL requires approximately 54% bilateral upper limb use (Van Delden et al. 2009). The upper limb also serves as an individual’s most functional extremity (Brukner et al. 2012; Pollock et al. 2014). Postural stability in the upper trunk is important for the ability to perform optimal upper limb movement, as it constitutes the link between the trunk and upper limb. Reduced postural stability of the upper trunk interrupts the transfer of energy, thus negatively influencing functioning of the upper limb (Aytar et al. 2012; Brukner et al. 2012). The ability to move the hand during functional activities requires dynamic stability of the proximal joints, including the upper limb, shoulder girdle and trunk (Hunter & Chrome 2002).

Postural stability can be divided into static and functional stability; however, achieving optimal postural stability remains complicated (Pickerill & Harter 2011) as an individual can present with a variety of impairments that affect optimal postural stability post-stroke. Postural stability requires a complex interaction of the stabilisers of the spine (the muscles), structural stability (the vertebral column), neural control and other components such as joint range of movement, trunk flexibility, muscle properties and biomechanical relationships amongst body segments that act together for the execution of ADL (Okada, Huxel & Nesser 2011; Shumway-Cook & Woollacot 2012).

The assessment and treatment of muscles of the shoulder should be included in postural stability of the upper trunk as they play an integral role in the transfer of forces across the body (Zazulak et al. 2007). This is important in order to be able to (1) maintain correct alignment and positioning, (2) remain stable during position changing, (3) execute ADL and (4) maintain mobility (Aydoğ et al. 2006; Karatas et al. 2004).

To maintain postural stability, the body must be able to integrate both sensory and motor processing and biomechanical strategies with learned responses to anticipate postural changes. Furthermore, the upper trunk should be able to control and adapt during internal and external changes of the body, including movement of the upper limb and balance challenges (Shumway-Cook & Woollacot 2012). The active sub-system of trunk muscles contributes to stability through co-contraction (Gardner-Morse & Stokes 2003; Van Dieën, Cholewicki & Radebold 2003).

The nervous system assists by controlling muscle activity that contributes to stability aided by a feedback system, which consists of sensory (muscle and joint receptors), visual and vestibular input (Goodworth & Peterka 2009; Moorhouse & Granata 2007). Thus, the neuromuscular system is important in maintaining postural stability by activating muscles during activities. During fast upper limb movements, muscle activation starts in the lower extremities and continues upwards through the trunk to the upper limb (Zazulak et al. 2007). Shumway-Cook and Woollacot (2012) explained that functional activities need patterns of joint stability and mobility throughout the body. If postural stability post-stroke is not regained and maintained, the patient may struggle with the execution of all distal movements and ADL (Pollock et al. 2014).

Despite many therapeutic modalities and approaches, the rehabilitation of the hemiplegic upper limb remains a challenge (Kwakkel, Kollen & Krebs 2008; Page et al. 2008; Thielman & Bonsall 2012). The lack of positive findings regarding outcomes of stroke rehabilitation can be ascribed to many reasons, such as the low statistical power of studies, the heterogeneity of study populations and the limited response to outcome measures (Van der Lee et al. 2001).

Treatment modalities that are available for the relearning of upper limb function include task-oriented training, passive movements, compensatory training, bilateral upper limb training, rhythmical auditory cueing combined with repetitive reaching, constraint-induced therapy, sensorimotor stimulation, weight-bearing and dynamic, high-intensity resistance training and mirror therapy (Brewer et al. 2012; Pattern et al. 2006; Stoykov, Lewis & Corcos 2009). Closed-chain weight-bearing exercises on the affected limb may also be used in order to try and facilitate normal movement patterns through correct biomechanical alignment and muscle activation, especially when the stroke survivor has poor activity because of hemi-neglect or dyspraxia (Bakhtiary & Fatemi 2008; Davies 2000; Lang et al. 2012).

Weight-bearing exercises have positive effects in all stages of recovery and should commence as early as possible (Lang et al. 2012). Targeted weight-bearing can be used to activate muscle activity, increase stability, normalise tone, maintain muscle length and provide sensory input to the involved side through proprioceptive stimulation (Davies 2000; Lang et al. 2012).

Weight-bearing is a dynamic process during which the patient is taught to activate muscles in the trunk by moving the body weight over from the stable upper limb, either by using side-sitting or puppy-prone position (Davies 2000; Lang et al. 2012). Muscles in both the affected and unaffected upper limb lengthen and shorten to maintain the upper limb on the support surface during trunk movements in a weight-bearing position. Use of the affected upper-limb for weight support does not require fine motor control, therefore patients with severe weakness and loss of motor control can learn to use their hemiplegic arm to support their body weight.

Weight-bearing on an extended arm (long-lever) is more difficult and requires active control of the elbow and wrist joints and postural stability of the upper trunk. Long-lever weight-bearing can be used with patients who have more selective control and is often applied in sitting with the affected arm bearing weight through an extended elbow with the arm at the side of the body (Davies 2000; Lang et al. 2012). Weight-bearing training should be used in conjunction with other therapy programmes for the upper limb including open-chain exercises such as reaching, dressing, grooming and eating, as upper limb function involves a number of open kinetic chain activities. Therefore, therapy should include both closed and open-chain weight-bearing training as the foci will be on the postural stability of the upper trunk and functional retraining (Bakhtiary & Fatemi 2008; Lang et al. 2012).

The Biodex© may be used to objectively measure an individual’s ability to stabilise the involved joints (Karimi et al. 2008), ensuring that adequate closed and open-chain weight-bearing exercises are prescribed to retrain functionality. The Biodex© was introduced in neurological rehabilitation in the early 2000s (Cachupe et al. 2001) and the programmes are used for the restoration of the affected motor skills by retraining new neural pathways, proprioception and the maintenance of positioning, balance and weight transfer. The Biodex© allows the patient to repeat the movements correctly and treatment session results are stored and assist in monitoring progress data objectively (Cachupe et al. 2001). The Biodex© has an alternative application for closed-chain scapular stabilisation exercises (Blackburn & Guido 2000) and targets the somatosensory and neuromuscular systems for maintenance of positioning, balance and weight transfer. The Biodex© also targets stability so as to improve and maintain control of the centre of gravity over the patient’s base of support (Cachupe et al. 2001; Karimi et al. 2008).

Limited research has been carried out on the Biodex© specifically with regard to its effect on the rehabilitation of postural stability in the upper trunk post-stroke. Studies have focused on lower limb stability and have found that during stance/bilateral weight-bearing the ability to maintain the centre of gravity can be addressed to improve the stability of weight-bearing joints and balance (Blackburn & Guido 2000; Karimi et al. 2008). Studies performed include the effect of Biodex© training on balance of patients who are neurologically impaired, the elderly and sports participants (Aydoğ et al. 2006; Baldwin, Thomas & Ploutz-Synder et al. 2004; Ballard 2005). Other studies have determined the Biodex©’s effect on coordination, proprioception, the neuromuscular system, stability training post-operatively, stability and retraining of the hip, knee and ankle joint abnormalities or injuries (Blackburn & Guido 2000; Cachupe et al. 2001; Karimi et al. 2008; Kovaleski et al. 2009; Pereira et al. 2008). Although evidence is widely available on the efficacy of the Biodex© on general populations and on patient outcomes post-stroke with regard to balance and gait retraining, there is a dearth of evidence on its effect on the hemiplegic upper limb (Aydoğ et al. 2006; Baldwin et al. 2004; Ballard 2005; Pereira et al. 2008).

The Biodex© consists of a multiaxial system that is used to objectively measure balance and postural stability on both a stable and an unstable base of support (Cachupe et al. 2001; Pereira et al. 2008). It provides immediate visual feedback on the patient’s ability to control their centre of gravity and assesses the neuromuscular control in closed-chain and multiplane exercises and assisting patients to relate to and repeat movements. It also renders progression and documents treatment sessions (Ballard 2005; Cachupe et al. 2001). Apart from assessing static and/or dynamic balance, the Biodex© also compares the involvement of bilateral effects of affected limbs (Ballard 2005; Cachupe et al. 2001).

High correlations have been found between increased weight-bearing and muscle activity in the shoulder in a weight-bearing position in sports participants utilising the Biodex© (Cachupe et al. 2001; Pereira et al. 2008). Weight-bearing is a central treatment modality of the affected upper-limb post-stroke in order to facilitate normal movement patterns through correct biomechanical alignment and muscle activation (Bakhtiary & Fatemi 2008). Even though weight-bearing exercises have been performed on the Biodex© for the affected lower-limb post-stroke none have been performed on the affected upper-limb. Weight-bearing over the hemiplegic side can be used to activate muscle activity, increase stability, normalise tone, maintain muscle length and provide sensory input to the involved side through proprioceptive stimulation (Davies 2000; Lang et al. 2012). Weight-bearing exercises may be used to reduce the risk of injury as joint compression and approximation act to enhance muscular co-contraction about the joint-producing dynamic stability (Bakhtiary & Fatemi 2008). The objective of using the Biodex© is to facilitate normal movement patterns by applying approximation through the weight-bearing limb. Based on the reported effects of the Biodex© on muscle activation with weight-bearing on the lower limbs, the authors applied the Biodex© as a treatment modality post-stroke to facilitate muscle activity around the shoulder in a closed-chain weight-bearing position. Our pilot study, therefore, aimed to determine the effect of the Biodex© as an added treatment modality, on the postural stability of the upper trunk post-stroke in a closed-chain weight-bearing position.

## Method

This was a longitudinal randomised control pilot trial (RCT) with single blinding of the research assistant. Participants were assigned randomly to one of two groups using computer-generated random numbers through concealed allocation (Altman 1991). The study population from which the study sample was drawn included all stroke patients admitted to the Life Rehabilitation Unit (Pasteur Hospital) in Bloemfontein from January 2014 to March 2015. Inclusion criteria were patients who had a stroke that resulted in hemiplegia and shoulder instability and were between the ages of 18 and 85 years. We excluded 130 participants based on the exclusion criteria described in Table 1.

**TABLE 1:** Reasons for excluding possible candidates from study (*n* = 130).

Exclusion criteria	Screening tool used	Indication for exclusion	*n*	%
Medically unstable	Medical examination by rehabilitation team doctor	Doctor recommended discontinuation of active rehabilitation	10	7.69
Extreme shoulder instability and/or pain	Screening by the first author	Positive Sulcus sign	1	0.77
		Intolerance of any passive or active movement with the affected upper limb	1	0.77
Cognitive impairment (all possible participants were screened for all 3 components)	Functional independence measure (FIM)	Less than 4/7 for the ‘problem solving’ component	111	85.38
		Less than 4/7 for the ‘memory’ component	111	85.38
	Mini-mental cognitive screen	Less than 15/30 on the mini-mental	111	85.38
Visual impairment	Screening by the first author	Inability to read a font size of Arial 12	6	4.62
Aphasia	Screening by the first author	Inability to speak	94	72.31
Other	Screening by the first author	Less than 3 weeks in rehabilitation	18	10.00
		No involvement of the upper limb	3	2.31
		Discharged themselves during the research period	2	1.54

The Biodex© consists of a circular platform that is able to move in different axis (anterior/posterior and medial/lateral) simultaneously, whilst adjusting stability over 12 levels (level 12 most stable = static; level 0 most unstable). The Biodex© provides a stability index (SI) value, which represents the displacement from a level platform position (0.0) in different motions, namely anterior/posterior, medial/lateral and overall (Hinman 2000). The overall SI takes into account the displacement from the level platform position in the anterior/posterior (sagittal plane) and the medial/lateral (frontal plane). The greater the variance, the poorer the neuromuscular response. A low score is an indication of better stability and postural stability of the upper trunk, as well as more stability of the surrounding and weight-bearing joints, whilst a high score is an indication of poor stability (Cachupe et al. 2001; Pereira et al. 2008). A high SI is associated with an unstable posture and indicates decreased postural stability of the upper trunk, whilst a low SI (closer to 0.0) is associated with a stable posture and indicates less postural instability of the upper trunk (Hinman 2000). The SI is calculated by using standardised formulae for different motions of movement (Cachupe et al. 2001; Pereira et al. 2008). The Biodex© Postural Stability test specifically focuses on patients’ ability to maintain their centre of balance and assesses deviations from the centre and indicates the overall SI, anterior/posterior index, medial/lateral index, the percentage time in each zone and the percentage time in each quadrant.

The reliability of postural stability testing using the Biodex© in patients could not be confirmed (Pickerill & Harter 2011). However, in a study conducted on the reliability of the Biodex©, the results across a series of eight trials showed that the Biodex© produces reliable measures on dynamic balance in healthy adults (Cachupe et al. 2001). The overall SI ranged between 0.90 and 0.94, anterior/posterior SI ranged between 0.86 and 0.95 and the medial/lateral SI range between 0.76 and 0.93. Kovaleski et al. (2009) also found highly reliable results assessing postural stability with SI values (anterior/posterior) ranging from 0.90 to 0.96.

Demographic information was obtained using a self-developed demographic data sheet.

## Ethical consideration

The necessary permissions were obtained from stakeholders prior to conducting the study. Ethical clearance for this study was obtained from both the Human Research Ethics Committee of the University of the Witwatersrand (M130405) and the University of the Free State (79/2013). Written permission was also obtained from the Manager of Life Rehabilitation unit (Pasteur Hospital) in Bloemfontein. All participants gave informed consent prior to the start of the study.

Both the control and experimental groups continued with the standard therapy programme at the centre. The standard therapy programme consisted of an interdisciplinary rehabilitation approach including a minimum of 3 hour and 30 min intensive therapy per day of physiotherapy, occupational therapy, speech therapy, social work and neuropsychology therapy. The individualised physiotherapy programme consisted amongst other things, of neuromuscular re-education, therapeutic exercises, mobilisation of joints, balance retraining, re-education of gait, task-specific training, retraining of ADL and improvement of problem-solving and motor-learning skills. Programmes at this centre aim to facilitate optimal functional independence and are individualised according to each patient’s needs (Life Healthcare 2015).

Both groups were tested at baseline and after the 1 month intervention by a research assistant, using the Biodex© and postural stability tests were performed at three levels of stability: 12 (maximum stability = static), 6 (moderate stability) and 1 (no stability). The postural stability test focuses on the patient’s ability to maintain her or his centre of balance and assesses deviations from the centre.

The experimental group also received additional postural stability (upper limb weight-bearing) training for the upper trunk using the Biodex© provided for by the first author. This intervention programme was designed by taking into consideration the FITT principles with regard to frequency, intensity and time and the type of exercise (Billinger et al. 2015). The clinical intervention on the Biodex© consisted of nine treatments distributed over a 3-week period. Each treatment lasted between 15 and 20 min, depending on the time taken to rest between exercises. Studies carried out by Billinger et al. (2015) and Gordon et al. (2004) suggested shorter therapy sessions post-stroke, starting with 10 min of continuous exercise, benefits can include limiting post-stroke fatigue or overexertion, preventing risk of using compensatory movement patterns and limiting the risk of injuring the limb or muscles. With each treatment the participant was placed in the correct predetermined position on the Biodex©, namely they were placed on a high–low Bobath plinth in puppy prone position, with elbows placed on the Biodex© at a 90° angle, with the shoulders perpendicular to the trunk. The screen of the Biodex© was adjusted so that the participant was able to see the screen for visual feedback. The first author ensured that the participant was in the correct posture to achieve optimal stability and safety. Two different interactive game-like training modes were used on the Biodex© during the treatment, namely weight-shift training and percentage weight-shift training.

Data analysis was undertaken using STATA, with statistical significance set at *p* < 0.05 (two-sided). Because of the small study population the data distribution was not normal for the intervention part of the study and non-parametric tests were used for the data analysis. For the pre- and post-intervention comparison in each group, the Mann–Whitney test was used to determine differences in the upper trunk postural stability. All demographic information was summarised using descriptive statistics. An intention-to-treat analysis was used.

## Results

A total of 15 participants were included in the study (eight in the experimental group and seven in the control group). The demographic details and the clinical characteristics of the study participants are shown in Table 2. The age of the participants ranged between 32 and 80 years, with a median age of 55 years.

**TABLE 2:** Socio-demographic and clinical characteristics of the study sample (*n* = 15).

Characteristics	Total study sample (*n* = 15)		Control group (*n* = 7)		Experimental group (*n* = 8)	
	*n*	%	*n*	%	*n*	%
**Gender**						
Male	8	53.33	2	28.57	6	75.00
Female	7	46.67	5	71.43	2	25.00
**Median Age**						
Male	53	13.85	60.5	27.58	53	10.35
Female	55	11.32	53	13.56	55.5	0.71
**Side of body affected**						
Left-sided hemiplegia	7	46.67	3	42.86	4	50.00
Right-sided hemiplegia	8	53.33	4	57.14	4	50.00
**Stroke subtype**						
Haemorrhage	1	0.67	1	14.29	0	0.00
Infarct	7	46.67	4	57.14	3	37.50
Not specified	7	46.67	2	28.57	5	62.50
**Employment**						
Employed	14	93.33	6	85.71	8	100.00
Unemployed	0	0.00	0	0.00	0	0.00
Pensioner†	1	0.67	1	14.29	0	0.00
**Other medical history**						
Heart disease	2	13.33	0	0.00	2	25.00
Hypertension	10	66.67	4	57.14	6	75.00
Diabetes	6	40.00	2	28.57	4	50.00
Cholesterol	4	26.67	1	14.29	3	37.50
Anticoagulants use	0	0.00	0	0.00	0	0.00
Hormone therapy	1	0.67	0	0.00	1	12.50
Smoking	0	0.00	0	0.00	0	0.00
Alcohol abuse	0	0.00	0	0.00	0	0.00

Note: Median age for the total study sample = 55 (interquartile range [IQR] = 17), range 32–80 years; for the control group = 55 (IQR = 32), range 32–80 years; for the experimental group = 53 (IQR = 24), range 45–69 years.

† , For the purpose of this study a pensioner was a retired person older than 60 years.

The control group comprised of two female and six male participants, whilst the experimental group comprised of five female and two male participants. The number of left-sided hemiplegics was similar to the number of right-sided hemiplegics. Some participants presented with multiple risk factors for stroke, with hypertension (66.67%) being the most frequently noted risk factor, followed by diabetes (40%) (Table 2).

The computerised scores for upper trunk postural stability at baseline and post 1 month intervention are shown in Table 3.

**TABLE 3:** Postural stability upper trunk at baseline and 1-month post-baseline (*n* = 15).

Variable	Baseline					1 month				
	Control group (*n* = 7)		Experimental group (*n* = 8)		*p*	Control group (*n* = 7)		Experimental group (*n* = 8)		*p*
	*n*	%	*n*	%		*n*	%	*n*	%	
**Overall**										
Level 12	3.05	1.4	1.49	1.04	0.610	0.76	0.51	0.43	0.54	0.28
Level 6	0.80	0.37	0.47†	0.24	0.020*	0.32	0.45	0.29	0.15	0.46
Level 1	0.78	0.79	0.50	0.39	0.190	0.36	0.29	0.43	0.18	0.61
**Anterior or Posterior**										
Level 12	2.13	1.41	1.02	0.77	0.340	0.49	0.37	0.31	0.37	0.34
Level 6	0.66	0.31	0.30†	0.17	0.009*	0.26	0.38	0.22	0.12	0.34
Level 1	0.72	0.65	0.39	0.25	0.190	0.27	0.19	0.31	0.14	0.34
**Medial or Lateral**										
Level 12	0.88	0.67	0.72	0.55	0.610	0.76	0.38	0.21	0.35	0.34
Level 6	0.50	0.22	0.22	0.14	0.150	0.16	0.23	0.15	0.09	0.69
Level 1	0.29	0.33	0.22	0.27	0.460	0.18	0.18	0.17	0.12	0.69

† , A mean value closer to 0.0 indicates a better value for upper trunk postural stability.

* , *p* < 0.05.

No statistically significant difference was found in the improvement of upper trunk postural stability between the control and experimental groups at 1-month post-baseline for any of the three levels tested. The overall SI (0.02) and the anterior/posterior SI (0.009) indicated significantly lower values in the experimental group at baseline on level 6.

Table 4 is a summary of the reference intervals (RIs) for the entire group at all three planes of movement, as well as the three levels of stability. Reference intervals are the range of values between the highest SI and lowest SI value (Hinman 2000). A RI closer to the (0, 0) SI represents a more stable upper trunk postural stability and level 6 had the smallest range for RI.

**TABLE 4:** Reference intervals for postural stability upper trunk in all three planes of movement (*n* = 15).

Variable	Overall	Anterior/Posterior	Medial/Lateral
**All participants**			
Level 12 (Static)	0.37–3.74	0.26–4.09	0.12–1.81
Level 6	0.21–1.52	0.11–1.26	0.07–0.77
Level 1 (Unstable)	2.31–0.18	1.88–0.09	0.11–0.90
**Control group**			
Level 12 (Static)	0.37–3.71	0.26–4.09	0.15–1.75
Level 6	0.27–1.52	0.24–0.24	0.07–0.77
Level 1 (Unstable)	0.22–2.31	0.17–1.88	0.11–0.88
**Experimental group**			
Level 12 (Static)	0.51–3.74	0.36–2.79	0.12–1.81
Level 6	0.21–0.81	0.11–0.60	0.11–0.51
Level 1 (Unstable)	0.18–1.39	0.09–0.88	0.11–0.90

## Discussion

Our results suggest that close-chain weight-bearing exercises post-stroke might be most beneficial when performed on a moderately unstable base, such as level 6 on the Biodex©. Performing an exercise on an unstable surface leads to greater muscle activity in an attempt to achieve greater stability (Behm, Anderson & Curnew 2002; Sandhu, Mahajan & Shenoy 2008). For this reason, better co-contraction of muscles (surrounding the upper trunk) might have occurred in participants during an increase in the movement of the platform. The activity of the antagonists will also increase on an unstable base of support in an attempt to control the position of the limb to prevent injuries (Pattern et al. 2006). However, if the base of support becomes too unstable, the control of the co-contraction is possibly of poorer quality, with resultant uncoordinated muscle activity. During level 12 (stable) throughout the study (baseline and 1-month post-test), the values are furthest away from the expected 0.0 indicating the clinical value of exercising on an unstable base rather than a stable base to elicit muscle co-contraction around a joint in a weight-bearing position. Values for the unstable base (level 1) throughout our study are further away from the expected 0.0 than on a moderate unstable base (level 6) confirming the negative clinical effects of weight-bearing exercises on a too unstable base of support.

From a biomechanical perspective, several mechanisms such as postural control and different platform placements allow the maintenance of postural stability on a moving surface (Pollock et al. 2000). Postural control requires highly complex interactions from both the sensory and motor nervous systems and is closely related to postural adjustment strategies during movement. Different types of platform displacements result in various proprioceptive and vestibular signals and muscle responses (Szturm & Fallang 1998). During platform movement, the body’s base of support moves to a new position and postural adjustment should take place to restore the centre of mass, which is controlled by the central nervous system. The postural adjustments may be made by more than one muscle, and the muscle may contract concentrically or eccentrically. To maintain postural stability, the body must be able to integrate both sensory and motor processing and biomechanical strategies with learned responses to be able to anticipate postural changes. The trunk should be able to control and adapt during internal and external changes of the body, including movement of the distal extremities and balance challenges (Pavol and Pai 2002; Shumway-Cook & Woollacot 2012) indicate that feedback (reactive) strategies are combined with feedforward (predictive) strategies to facilitate postural adjustment/compensation during movement. Postural responses to movement are flexible and dependent on the activity (Scholz et al. 2007). This may provide a possible explanation for why smaller ranges were achieved for the RI at level 6, as the small degree of movement allowed for muscular and sensory interaction.

It may be valuable for therapists using the Biodex© (or planning to procure a Biodex©) for post-stroke rehabilitation of the upper trunk postural stability to perform close-chain weight-bearing exercises on level 6 (moderately unstable) rather than level 1 (unstable) or level 12 (stable). These results may inform therapists who are not using the Biodex© (because of the high financial output procuring a Biodex©) to consider exercising patients post-stroke in a moderately unstable weight-bearing position. These suggestions for clinical practice once again highlight the importance of co-contraction and the postural responses that need to be re-educated post-stroke to enhance postural trunk stability.

This was a pilot study with obvious limitations. It was difficult to recruit patients who could tolerate the Biodex© and the measurement positions needed for the execution of the study. The suitability of the Biodex© in upper trunk postural stability training, therefore, is questionable. In addition, the cost of the Biodex© makes its widespread use in low- and middle-income countries unlikely. However, it should be noted that, given a larger sample size and measurements over a longer period of time, different results could possibly have been found. The differences in the level 6 stability values at baseline may have been because of the small sample size.

Both groups also received standard rehabilitation and the Biodex© was only an adjunct intervention strategy for the experimental group. Thus, any change in the outcome of upper trunk postural stability cannot be attributed directly and solely to the Biodex© as there were no differences in the changes in postural stability between the two groups over the study period with both groups improving. This indicates that rehabilitation post-stroke should include activities that enhance co-contraction of muscles and the postural responses that need to be re-educated post-stroke to enhance postural trunk stability (Prange et al. 2006; Shadish, Clark & Steiner 2008; Sze et al. 2002).

## Conclusion and recommendations

Upper limb weight-bearing training (using the Biodex©) had no statistically significant effect on the upper trunk postural stability in patients with hemiplegia post-stroke in comparison with the control group. However, it is noteworthy that the RI at level 6 achieved smaller ranges, and this could be further investigated in larger post-stroke upper-limb studies.

The authors aimed at identifying an additional treatment modality for the rehabilitation of the upper limb post-stroke in this pilot study. Although evidence-based therapeutic modalities for and approaches to rehabilitating the upper limb post-stroke do exist, no one modality has been shown to be more effective than another. Our study may provide a basis for the development of further physiotherapy intervention programmes using the Biodex© as an additional and innovative tool in upper trunk postural stability rehabilitation. It must be noted, however, that the cost of the Biodex© makes this equipment unsuitable for use in the public sector where the majority of our patients are treated. Thus, other methods of achieving postural stability must be considered.
